# Mismatch Negativity as a “Translatable” Brain Marker Toward Early Intervention for Psychosis: A Review

**DOI:** 10.3389/fpsyt.2013.00115

**Published:** 2013-09-23

**Authors:** Tatsuya Nagai, Mariko Tada, Kenji Kirihara, Tsuyoshi Araki, Seiichiro Jinde, Kiyoto Kasai

**Affiliations:** ^1^Department of Neuropsychiatry, Graduate School of Medicine, University of Tokyo, Tokyo, Japan; ^2^Department of Youth Mental Health, Graduate School of Medicine, University of Tokyo, Tokyo, Japan

**Keywords:** mismatch negativity, early intervention, first-episode psychosis, high-risk for psychosis, schizophrenia, animal, translatable

## Abstract

Recent reviews and meta-analyses suggest that reducing the duration of untreated psychosis leads to better symptomatic and functional outcome in patients with psychotic disorder. Early intervention attenuates the symptoms of individuals at clinical high-risk (HR) for psychosis and may delay or prevent their transition to psychosis. Identifying biological markers in the early stages of psychotic disorder is an important step toward elucidating the pathophysiology, improving prediction of the transition to psychosis, and introducing targeted early intervention for help-seeking individuals aiming for better outcome. Mismatch negativity (MMN) is a component of event-related potentials that reflects preattentive auditory sensory memory and is a promising biomarker candidate for schizophrenia. Reduced MMN amplitude is a robust finding in patients with chronic schizophrenia. Recent reports have shown that people in the early stages of psychotic disorder exhibit attenuation of MMN amplitude. MMN in response to duration deviants and in response to frequency deviants reveals different patterns of deficits. These findings suggest that MMN may be useful for identifying clinical stages of psychosis and for predicting the risk of development. MMN may also be a “translatable” biomarker since it reflects *N*-methyl-d-aspartte receptor function, which plays a fundamental role in schizophrenia pathophysiology. Furthermore, MMN-like responses can be recorded in animals such as mice and rats. This article reviews MMN studies conducted on individuals with HR for psychosis, first-episode psychosis, recent-onset psychosis, and on animals. Based on the findings, the authors discuss the potential of MMN as a clinical biomarker for early intervention for help-seeking individuals in the early stages of psychotic disorder, and as a translatable neurophysiological marker for the preclinical assessment of pharmacological agents used in animal models that mimic early stages of the disorder.

## Introduction

Recent reviews and meta-analyses suggest that patients with shorter duration of untreated psychosis (DUP) show better symptomatic and functional outcome (1–3). For example, early detection in first-episode psychosis (FEP) leads to a higher percentage of recovery over 10 years relative to usual-detection patients (4). Furthermore, early intervention in individuals with clinical high-risk (HR) for psychosis attenuates their symptoms and potentially delays or prevents their transition to psychosis (5–9). These findings suggest that early detection and intervention play a critical role in the improvement of functional outcome and even in the prevention of psychosis.

High-risk individuals can be identified using clinical criteria based on symptomatology (10); the rate of transition to psychosis is approximately 35% within 2–3 years of follow-up (11), which is substantially higher than the incidence rate of psychosis in the general population. Higher sensitivity and specificity are required from the viewpoint of targeted early intervention.

For this reason, identifying biological markers in the early stages of psychotic disorders is an important step not only toward elucidating the underlying pathophysiology but also toward improving prediction of the transition to psychosis and introducing targeted early intervention to help-seeking individuals aiming for better outcome (12–14).

Auditory mismatch negativity (MMN) is a component of the event-related potential (ERP) and a promising biomarker candidate for psychotic disorders such as schizophrenia. A meta-analysis and several reviews of MMN in chronic schizophrenia (CSZ) are currently available (15–21). In this article, we focus on MMN in the early stages of psychotic disorders.

Mismatch negativity may also be a “translatable” biomarker because MMN reflects *N*-methyl-d-aspartte (NMDA) receptor function which plays a fundamental role in the pathophysiology of schizophrenia (22, 23), and MMN-like response can be recorded in animals including mice and rats (described later).

The following sections review MMN studies conducted on individuals in the early stages of psychosis and also on animals. In particular, the authors pay special attention to the finding that MMN in response to duration deviant stimuli and MMN in response to frequency deviant stimuli demonstrate different characteristics in the early stages of psychosis. Based on the results of these studies, the authors discuss the potential of MMN as a clinical biomarker for early intervention in help-seeking individuals and also as a translatable neurophysiological marker for the preclinical assessment of pharmacological agents tested in animal models that mimic early stages of the disorder.

## General Background of MMN

Mismatch negativity is an ERP component elicited when an infrequent deviant stimulus occurs in a sequence of repetitive auditory stimuli. In an oddball paradigm, deviant stimuli differ from repetitive standard stimuli in one or more perceptual characteristics, including frequency, duration, intensity, location, spectro-temporal pattern, and phonemes (16, 19). MMN is even elicited under passive conditions when subjects ignore the stimuli. Thus, it is considered an index of preattentive auditory discrimination as well as a preattentive form of sensory memory (24).

**Figure 1:**
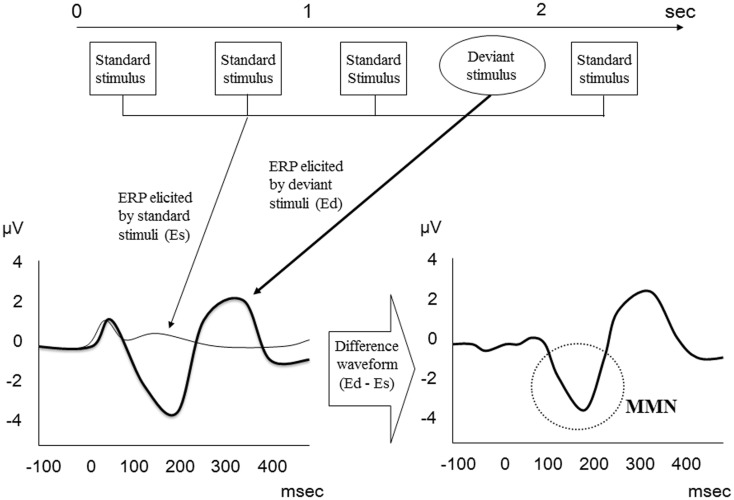
**MMN waveform**. MMN relates to the difference wave obtained by subtracting the standard stimulus ERP from the deviant stimulus ERP.

Mismatch negativity relates to the difference wave obtained by subtracting the standard stimulus ERP from the deviant stimulus ERP (Figure 1) and usually peaks between 150 and 250 ms after presentation of the deviant stimulus (24). On electroencephalogram (EEG), maximal MMN responses are evident at frontocentral scalp recording sites, with phase reversal at mastoids.

## MMN in Chronic Schizophrenia

Reduced MMN amplitude is one of the most robust findings in schizophrenia (25), and the mean effect size is approximately 0.99 (17). Given its high test-retest reliability (26), MMN has been proposed as a statistically reliable biomarker for schizophrenia.

Although many studies have used duration and/or frequency as deviant stimuli in auditory oddball paradigms, MMN in response to duration deviants (dMMN) and in response to frequency deviants (fMMN) have different sensitivity. Michie et al. (15) examined both duration and frequency deviants in CSZ patients and demonstrated that amplitude reduction is larger in dMMN than in fMMN. Meta-analysis conducted on CSZ patients also revealed that the effect size of dMMN is larger than fMMN (17).

Mismatch negativity amplitude reduction reflects sensory network dysfunction in schizophrenia, as attention and motivation have little effect on MMN (15, 16, 19, 24). This provides an important advantage in clinical settings since patients are not required to perform an active task.

Previous studies have shown that antipsychotic medication has little effect on MMN (27–32); however, recently Zhou et al. (33) reported that antipsychotics such as aripiprazole improve MMN amplitude reduction in schizophrenia. Benzodiazepine has been reported to have no significant effect on MMN amplitude (34). Interestingly, it has been suggested that drugs acting at the NMDA receptor may have a significant effect on MMN in schizophrenia. Lavoie et al. (35) reported that *N*-acetyl-cysteine, a glutathione precursor that can potentiate the activity of NMDA receptors, increases fMMN amplitude in schizophrenia patients. However, further studies are needed to clarify whether other modulators of NMDA receptors, such as glycine transporter inhibitors and d-serine, can similarly enhance MMN amplitude in schizophrenia.

Correlations between MMN amplitude and clinical variables have been described in the literature. For example, it has been reported that the amplitude of dMMN is associated with social function (36), social cognition (37), and executive function (38) in CSZ, while phonetic MMN amplitude has been reported to be associated with verbal memory (39) and social skills acquisition (40). These findings indicate that MMN is a biologically and clinically significant index of schizophrenia.

## MMN in Patients with First-Episode or Recent-Onset Psychosis

In recent years, the number of MMN reports on FEP and on recent-onset psychosis (ROP) has grown (see Table 1). These studies suggest that dMMN and fMMN have different properties, as seen in CSZ.

**Table 1:** **Demographic data of previous studies of FEP or ROP**.

Publication	HC			Patients				Comments
	N	M/F	Age	N	M/F	Age	DOI (y)	
Javitt et al. (41)	20	8/7	36.3 (9.5)	13 (RSZ)	10/3	27.4 (2.7)		Most patients took medication
Salisbury et al. (42)	27	20/7	24.2 (4.3)	21 (FES)	18/3	24.9 (6.2)		Most patients took medication
Oades et al. (43)	22	12/10	17.6 (0.4)	28 (FES)	21/7	17.5 (0.4)		
Umbricht et al. (44)	39	26/13	30.5 (7.1)	26 (FES)	19/7	23.9 (5.5)	0.05 (0.1)	Most patients took SGA
				26 (RSZ)	14/12	30.3 (6.7)	3.4 (1.5)	
Devrim-Ucok et al. (45)	34	19/15	24.5 (6.4)	30 (FES acute)	15/15	22.1 (5.7)		Ten acute FES patients took medication
				21 (FES post)	12/9	21.6 (5.6)		All post FES patients took medication
Magno et al. (46)	27	13/14	38.0 (12.9)	12 (FES)	9/3	24.3 (6.2)		Drug-naïve except for one patient taking chlorpromazine
Todd et al. (47)	14	7/7	24.0 (11.7)	14 (SZ short)	8/6	25.0 (10.7)	2.6 (1.7)	Most patients took SGA
Hermens et al. (48)	17	7/9	22.6 (2.8)	17 (FEP)	12/5	22.5 (3.2)		Patients; 1 SZ, 3 SZA, 5 SZP, 2 BP, 6 MDD
								Medication; 15 SGA, 9 AD, 3 mood stabilizers
Bodatsch et al. (49)	67	35/32	25.8 (4.0)	33 (FES)	26/7	26.0 (6.5)		
Jahshan et al. (50)	28	18/10	19.2 (3.4)	31 (RSZ)	25/6	29.8 (3.6)	1.2 (0.8)	Twenty-five patients took SGA
Kaur et al. (51)	18	11/7	23.1 (3.0)	17 (FEPa)	10/7	22.8 (4.6)		Most patients took SGA
				18 (FES)	13/5	22.2 (3.5)		
Atkinson et al. (12)	61	20/41	19 (3.5)	11 (FEP)	5/6	21 (2.7)		Eight patients took antipsychotics
Higuchi et al. (52)	20	14/6	25.4 (6.9)	20 (FES)	9/11	27.2 (7.3)	0.65 (0.5)	Seven patients had no medication
								More than half of the patients took SGA
Mondragon-Maya et al. (53)	24	14/10	22.6 (5.8)	20 (FEP)	13/7	26.1 (7.2)		Antipsychotic naive

All values are shown as mean (standard deviation).

AD, antidepressant; BP, bipolar disorder; DOI, duration of illness; FEP, first-episode psychosis; FEPa, FEP with affective-spectrum; FES, first-episode schizophrenia; HC, healthy controls; MDD, major depressive disorder; ROP, recent-onset psychosis; RSZ, recent-onset schizophrenia; SGA, second-generation antipsychotics; SZ, schizophrenia; SZA, schizoaffective disorder; SZP, schizophreniform disorder.

FES acute means patients with FES on acute phase.

FES post means patients with FES on post-acute phase when their symptoms improved.

SZ short means patients with schizophrenia who received their first diagnosis within 5 years.

All of these studies reported a significant attenuation of dMMN and fMMN amplitude in ROP, including recent-onset schizophrenia (RSZ), subjects compared to healthy controls (HC). Excluding the findings of Magno et al. (46), they also reported a significant attenuation of dMMN amplitude in FEP subjects compared to HC. In contrast, only Devrim-Ucok et al. (45) noted a significant reduction of fMMN amplitude in FEP subjects compared to HC (see Table 2). Salisbury et al. (54) reported that fMMN amplitude in first-episode schizophrenia (FES) patients became significantly smaller compared to HC approximately 1.5 years after onset of the illness, which correlated with a reduction in Heschl’s gyrus volume. Thus, fMMN may reflect progression of the disease pathology, and dMMN amplitude may be attenuated before the onset of psychosis.

**Table 2:** **Oddball paradigm and results of previous studies of FEP or ROP**.

Publication	Deviant	Stimulus characteristics				Probability (%)	ISI/SOA	Electrodes	Results of MMN amplitude
		Standard (Hz)		Deviant (Hz)	
		Frequency	Duration	Frequency	Duration	
Javitt et al. (41)	Duration	1000	100	1100	100	12.5		Fz	dMMN; HC > RSZ
	Frequency			1000	250	12.5	667–770	Fz	fMMN; HC > RSZ
Salisbury et al. (42)	Frequency	1000	100	1200	100	5	333	All and midline	fMMN; HC = FES
Oades et al. (43)	Duration	800	80	800	40	10	850–1050	FC	dMMN; HC > FES
	Frequency			600	80	10	850–1050		Only dMMN was analyzed
Umbricht et al. (44)	Duration	1000	100	1500	100	10	300	FC	dMMN; HC > FES and RSZ
	Frequency			1000	250	10	300	FC	fMMN; HC > RSZ, HC = FES
Devrim-Ucok et al. (45)	Frequency	1000	50	1500	50	20	1500	FCP	fMMN; HC = FESa, HC > FESp
Magno et al. (46)	Duration	1000	50	1000	25	10	500	FC	dMMN; HC = FES
	Frequency			1200	50	10	500	FC	fMMN; HC = FES
Todd et al. (47)	Duration	1000	80	1000	125	6	450	FC	dMMN; HC > SZ short
	Frequency			1200	80	6	450	FC	fMMN; HC = SZ short
	Intensity			1000	80	6	450	FC	iMMN; HC > SZ short
Hermens et al. (48)	Duration	1000	50	1000	100	8	500	Fz, Cz	dMMN; HC > FEP
Bodatsch et al. (49)	Duration	1000	80	1000	40	10	500 ± 150	FC	dMMN; HC > FES
	Frequency			1200	80	10	500 ± 150	FC	fMMN; HC = FES
Jahshan et al. (50)	Duration	1000	50	1000	100	10	500	FC	dMMN; HC > RSZ
Kaur et al. (51)	Duration	1000	50	1000	100	8	500	Fz, Cz	dMMN; HC > FEPa, HC > FES
Atkinson et al. (12)	Duration	1000	50	1000	100	7.5	600	Fz, Cz	dMMN (increment); HC > FEP
	Duration	1000	100	1000	50	7.5	600	Fz, Cz	dMMN (decrement); HC > FEP
Higuchi et al. (52)	Duration	1000	50	1000	100	10	500	Fz	dMMN; HC > FES
Mondragon-Maya et al. (53)	Frequency	1000	100	1500	100	10	300	FC	fMMN; HC = FEP

FC, frontocentral; FCP, frontocentral and parietal; HC, healthy controls; ISI, interstimulus interval; FEP, first-episode psychosis; FEPa, FEP with affective-spectrum; FES, first-episode schizophrenia; ROP, recent-onset psychosis; RSZ, recent-onset schizophrenia; SOA, stimulus onset asynchrony; SZ, schizophrenia.

FESa means patients with FES on acute phase.

FESp means patients with FES on post-acute phase when their symptoms improved.

SZ short means patients with schizophrenia who received their first diagnosis within 5 years.

“*A* > *B*” means that *A* is significantly larger than *B*. “*A* = *B*” means that *A* and *B* are not significantly different.

Within dMMN, the duration increment deviants (long duration) and the duration decrement deviants (short duration) may have different sensitivity. One study has demonstrated that a duration increment condition can discriminate between patients with CSZ and HC better than a duration decrement condition (16). While it is unclear whether this superiority of duration increment over duration decrement is true of FEP, it is interesting to note that all of the studies reporting a significantly attenuated dMMN in FEP or ROP utilized duration increments, whereas only Magno et al. (46) chose duration decrement and failed to show significantly decreased dMMN in FES (see Table 2). Atkinson et al. (12) examined both duration increment and duration decrement and found that MMN to both duration deviants was reduced in FEP compared to HC. Discrepancies in the results of Magno et al. (46) and Atkinson et al. (12) may be due to differences in sample characteristics and the methods used to measure MMN.

The effect of medication on MMN has not been sufficiently investigated in FEP and ROP, although the finding that medication has little effect on MMN in CSZ lends to the possibility that a similar effect may be observed in FEP and ROP. However, further studies are needed to clarify this point.

Todd et al. (47) reported a significant positive correlation between fMMN amplitude and total score of the Schedule for Assessment of Positive Symptoms (SAPS), Delusions, Positive Formal Thought Disorder, and between iMMN amplitude and Hallucination, which indicates that higher symptom severities were associated with smaller MMN amplitude. In contrast, no correlation was found between dMMN amplitude and SAPS. Other studies have reported that no significant correlation exists between MMN amplitude and positive symptoms, as assessed by the Brief Psychiatric Rating Scale (BPRS) or the SAPS (12, 42, 43, 45).

As to negative symptoms, Oades et al. (43) reported that reduced mastoid dMMN is related to anergia and flat affect, as assessed by the Schedule for Assessment of Negative Symptoms (SANS). Umbricht et al. (44) demonstrated that a larger fMMN is associated with a higher SANS total score. However, other studies have described no significant correlation between MMN amplitude and negative symptoms, as assessed by BPRS or SANS (12, 42, 45, 47).

Besides positive and negative symptoms, MMN amplitude has also been significantly associated with anxious depression factor (42), Clinical Global Impression (CGI) (43), and cognitive functions (48, 51). On the other hand, it has been reported that MMN amplitude does not correlate with DUP (45), duration of illness (DOI) (46), or the Global Assessment of Functioning (GAF) (50).

Although a number of studies have noted a correlation between MMN amplitude and various clinical ratings in FEP as well as in CSZ, the findings are relatively inconsistent. Further studies with a large sample size are needed to confirm these correlations. In addition, a cross-sectional design study may not be appropriate since fMMN shows a progressive decrease after the onset of psychosis. Longitudinal studies will be more useful for investigating the association between MMN and clinical variables.

## MMN in Individuals at Clinical High-Risk for Psychosis

Since Brockhaus-Dumke et al. (55) first examined MMN in individuals at clinical HR for psychosis, several MMN studies targeted at HR individuals have been reported (see Table 3). Regarding the criteria for HR, all of the studies utilized either the Bonn Scale for the Assessment of Basic Symptoms (BSABS) (56), Comprehensive Assessment of At-Risk Mental State (CAARMS) (57), or the Structured Interview for Prodromal Symptoms (SIPS) (58). CAARMS and SIPS include three subgroups for HR: attenuated psychotic symptoms (APS), brief limited intermittent psychotic episode (BLIP), and genetic risk and deterioration syndrome (GRD). BSABS describes basic symptoms (59).

**Table 3:** **Demographic data of previous studies of HR**.

Publication	HC			HR					Conversion to psychosis
	N	M/F	Age	N	M/F	Age	Criteria	Medication	
Brockhaus-Dumke et al. (55)	33	28/15	24.5 (3.3)	43	29/14	25.4 (5.8)	BSABS		
Shin et al. (60)	18	12/6	22.1 (2.0)	16	10/6	21.3 (3.2)	CAARMS	3 SGA	2 Individuals
Bodatsch et al. (49)	67	35/32	25.8 (4.0)	62	41/21	24.8 (6.0)	BSABS	None	25 (23 SZ, 1 SZP, 1 DD)
Jahshan et al. (50)	28	18/10	19.2 (3.4)	26	22/4	21.9 (3.7)	SIPS	7 SGA	2 (1 Manic, 1 SZ)
Atkinson et al. (12)	61	20/41	19 (3.5)	30	10/20	17 (3.6)	CAARMS	7 RIS	6 (3 SZA, 1 SZPa, 1 SZUn, 1 P-NOS)
Shaikh et al. (14)	50	25/25	24.6 (4.5)	41	26/15	24.7 (4.7)	CAARMS	None	10 (9 SZ, 1 Bipolar)
Higuchi et al. (52)	20	14/6	25.4 (6.9)	17	4/13	19.4 (4.4)	CAARMS	3 AP	4 (4 SZ)
Mondragon-Maya et al. (53)	24	14/10	22.6 (5.8)	23	16/7	20.1 (5.4)	SIPS	None	

All values are shown as mean (standard deviation).

AP, antipsychotics; BSABS, the Bonn scale for the assessment of basic symptoms; CAARMS, the comprehensive assessment of at-risk mental state; DD, delusional disorder; HC, healthy controls; HR, clinical high-risk for psychosis; P-NOS, Psychotic disorder not otherwise specified; RIS, risperidone; SGA, second-generation antipsychotics; SIPS, the structured interview for prodromal symptoms; SZ, schizophrenia; SZA, schizoaffective disorder; SZP, schizophreniform disorder; SZPa, schizophrenia paranoid type; SZUn, schizophrenia undifferentiated type.

The average observation period in these studies was approximately 7 months to 2 years, and the rate of conversion to psychosis was as follows: 13% [2/16; (60)], 40% [25/62; (49)], 8% [2/26; (50)], 20% [6/30; (12)], 24% [10/41; (14)], and 24% [4/17; (52)]. Most of these rates were consistent with a previous study that assessed nearly 300 HR help-seeking individuals whose conversion rate was approximately 35% within 2–3 years of follow-up (11).

Most of these HR-focused studies examined dMMN (see Table 4), which might be because dMMN amplitude attenuation has a higher sensitivity than fMMN in FEP and in CSZ. Three studies examined fMMN (49, 53, 55), and all of them failed to show reduced fMMN amplitude in HR compared to HC, which is consistent with the previous findings that fMMN reflects the progressive pathological process and that fMMN amplitude reduction is marked after the onset of psychosis (54). Significant reductions of dMMN amplitude were observed in most of the studies (12, 14, 50, 60). However, in two studies, dMMN amplitude in HR individuals was not significantly smaller than that observed in HC, and dMMN amplitude of converters to psychosis was significantly attenuated compared to HC (49, 52). Therefore, dMMN amplitude reduction seems evident as early as before the onset of full-blown psychosis.

**Table 4:** **Oddball paradigm and results of previous studies of HR**.

Publication	Deviant	Stimulus characteristics				Probability (%)	ISI/SOA	dB	Electrodes	Results of MMN amplitude
		Standard		Deviant	
		Frequency	Duration	Frequency	Duration	
Brockhaus-Dumke et al. (55)	Duration	1000	80	1000	40	10	500 ± 150	75	FC	dMMN; HC = HR
	Frequency			1200	80	10	500 ± 150	75	FC	fMMN; HC = HR
Shin et al. (60)	Duration	1000	50	1000	100	18.2	300	80		dMMN Dipole moment; HC > HR
Bodatsch et al. (49)	Duration	1000	80	1000	40	10	500 ± 150	75	FC	dMMN; HC = HR, HC > HR-C
	Frequency			1200	80	10	500 ± 150	75	FC	fMMN; HC = HR
Jahshan et al. (50)	Duration	1000	50	1000	100	10	500	85	FC	dMMN; HC > HR
Atkinson et al. (12)	Duration	1000	50	1000	100	7.5	600	70.5	Fz, Cz	dMMN (Increment); HC > HR
	Duration	1000	100	1000	50	7.5	600	70.5	Fz, Cz	dMMN (Decrement); HC > HR
Shaikh et al. (14)	Duration	1000	25	1000	50	15	300	80	Fz, F3, F4	dMMN; HC > HR
Higuchi et al. (52)	Duration	1000	50	1000	100	10	500	60	Fz	dMMN; HC = HR, HC > HR-C
Mondragon-Maya et al. (53)	Frequency	1000	100	1500	100	10	300		FC	fMMN; HC = HR

FC, frontocentral; HR, clinical high-risk for psychosis; HR-C, HR who converted to psychosis; ISI, interstimulus interval; SOA, stimulus onset asynchrony.

“*A* > *B*” means that *A* is significantly larger than *B*. “*A* = *B*” means that *A* and *B* are not significantly different.

Shin et al. (60) demonstrated that a smaller left dMMN magnetic counterpart dipole moment was associated with a larger positive symptom score, as measured by CAARMS. Other studies reporting on the association between MMN amplitude and clinical symptoms, as assessed by BPRS, SAPS, or SANS, have not described any significant relations. Although an association between dMMN amplitude and GAF was reported in CSZ, two studies that examined GAF and MMN in HR failed to find a significant relation (50, 60). As to the relation between MMN and cognitive function, Higuchi et al. (52) showed that a larger dMMN amplitude was associated with a larger score of verbal fluency, as assessed by the Brief Assessment of Cognition in Schizophrenia (BACS). On the other hand, Brockhaus-Dumke et al. (55) reported no relation with multiple domains of cognitive function.

Finally, dMMN might have the ability to predict the conversion from HR to psychosis. Bodatsch et al. (49); Shaikh et al. (14), and Higuchi et al. (52) reported that converters to psychosis have significantly reduced dMMN amplitudes at presentation relative to non-converters. Furthermore, Bodatsch et al. (49) showed that dMMN amplitude could predict onset of psychosis; a prognostic score was calculated based on a Cox regression model and stratified into two risk classes, which revealed significantly different survival curves. Previous studies with a large cohort of individuals at clinical HR have demonstrated that clinical variables such as clinical symptoms and social dysfunction can predict the onset of psychosis in multivariate prediction algorithms (11, 61). Thus, dMMN may improve the predictive power for onset of psychosis in HR individuals.

## MMN in Animals

MMN-like responses have also been reported in monkeys (62, 63), cats (64), guinea pigs (65), rats (66–75), and mice (76–78). Some studies have reported that evoked ERP responses are not necessarily MMN in rabbits (79), rats (80, 81), and mice (82).

Previous studies using animal models have shown that antagonists of NMDA receptors reduce MMN (22, 69, 77). Given that NMDA receptors play an important role in the pathophysiology of schizophrenia, MMN may be a biomarker of dysfunctional NMDA receptors in this disease. Ehrlichman et al. (78) reported that mutant mice heterozygous for neuregulin 1 showed reduced MMN. Since neuregulin 1 is one of the susceptibility genes for schizophrenia, MMN may be an intermediate phenotype that links genes to schizophrenia. These findings suggest that MMN may be useful for investigating molecular and cellular mechanisms of schizophrenia.

Recently, the neural adaptation hypothesis has been proposed as a general mechanism underlying MMN, which challenges the traditional sensory memory hypothesis (83). The former hypothesis argues that repeated presentation of standard stimuli results in an adapted and attenuated response of neurons in the auditory cortex, whereas rare deviant stimuli evoke a larger response of neurons that are less adapted. Thus, MMN might reflect a stimulus-specific adaptation (SSA) rather than genuine deviance detection. A number of studies have attributed MMN-like responses to SSA in monkeys (84), cats (85), rats (86–91), and mice (92).

In order to resolve the controversy between the two hypotheses, several studies have adopted a many standards control paradigm to differentiate between SSA and genuine deviance detection. Some studies support the deviance detection theory (73, 74, 88), while others do not (84). Although NMDA receptor antagonists have been shown to attenuate MMN amplitude (22, 69, 77), Farley et al. (88) reported that SSA is not affected by NMDA receptor antagonism and suggested that the NMDA sensitivity reported for the MMN might occur at a mechanistic locus outside of SSA. In other words, the SSA hypothesis cannot explain MMN.

Recent modeling studies have proposed that MMN reflects prediction rather than adaptation (93, 94). These studies found that the prediction error hypothesis based on Bayesian inference can explain the property of MMN measured in human subjects. To our knowledge, however, there is no animal study that has investigated MMN based on the prediction error hypothesis. Since it has been found that NMDA receptor antagonists alter the process associated with prediction error (95), this hypothesis may provide a neurobiological mechanism that links NMDA receptors to MMN.

Overall, compared to human studies, there are more inconsistencies in the MMN studies carried out on animals. Even if MMN-like responses are evoked, the polarity and latency window of responses vary in rats (74). These inconsistencies could result from species or line differences, anesthesia effect, stimuli or paradigm differences, and different cortical layers targeted.

Improved protocols and replication of studies might overcome these variables, after which utilization of MMN as a translatable brain marker could be feasible for the preclinical assessment of pharmacological agents in animal models that mimic the early stage of psychotic disorders.

## Conclusion

Mismatch negativity amplitude reduction is one of the most robust neurophysiological findings in schizophrenia patients. The amplitude of dMMN and that of fMMN have different characteristics. The fMMN amplitude may reflect the progressive pathological process and is attenuated after the onset of first-episode psychosis along with the reduction of Heschl’s gyrus volume. The dMMN amplitude reduces before the onset of psychosis and may be a significant predictor of the conversion to psychosis. Since early interventions may delay or prevent the transition to psychosis, dMMN may be useful for identifying people who require early intervention. In contrast, fMMN may be a potential therapeutic target for preventing the disease progression. Although further longitudinal studies are needed, MMN may be an important step toward introducing targeted early intervention of help-seeking people aiming for a better outcome.

Animal studies have shed light on the underlying cellular mechanisms of MMN. If further studies could clarify these molecular and cellular mechanisms then MMN could potentially be used as a translatable brain marker for the preclinical assessment of pharmacological components designed to improve symptoms and cognitive and/or functional impairment in individuals in the early stages of psychosis.

## Author Contributions

Tatsuya Nagai wrote the first draft of the manuscript. Mariko Tada, Kenji Kirihara, Tsuyoshi Araki, and Kiyoto Kasai discussed and revised the manuscript.

## Conflict of Interest Statement

The authors declare that the research was conducted in the absence of any commercial or financial relationships that could be construed as a potential conflict of interest.
